# Impact of COVID-19 pandemic on pediatric dental procedures in primary healthcare settings in Piracicaba, Brazil: an ecological study

**DOI:** 10.1186/s12875-024-02315-6

**Published:** 2024-02-27

**Authors:** Rúbia Vanessa Figueiredo dos Santos, Fernanda Maria Rovai Bado, Inara Pereira da Cunha, Marcelo de Castro Meneghim

**Affiliations:** 1 Piracicaba Dental School, University of Campinas, Piracicaba, Brazil; 2 Public Health School, Campo Grande, Mato Grosso do Sul, Brazil

**Keywords:** COVID-19, Primary health care, Unified health system, Oral health

## Abstract

**Background:**

The COVID-19 pandemic brought significant changes to dental care, which may have affected pediatric dental care offered in primary healthcare settings. Therefore, the aim of this study was to analyze the quantity of dental procedures performed in primary healthcare for children aged 6 to 12 years, before and during the COVID-19 pandemic.

**Methods:**

This is an ecological study using data from the health information system of Piracicaba, São Paulo, Brazil. The variables considered were: coverage of first programmed dental consultation, restoration of permanent and deciduous teeth, topical fluoride application (individual per session), emergency care, and deciduous tooth extraction. Two periods were considered: period I (March 1, 2019 to February 29, 2020) and period II (April 1, 2020 to March 31, 2021), before and during the pandemic, respectively. Comparisons between periods were made using the paired nonparametric Wilcoxon test with a significance level of 5%.

**Results:**

There was an increase in emergency care from 15.4 to 32.4% (*p* = 0.0095) and a decrease in the number of restorations of deciduous teeth from 32.8 to 20.2% (*p* = 0.0217). The first programmed consultation showed a decrease of 9.60% (*p* = 0.0930) in period II.

**Conclusions:**

The COVID-19 pandemic has hindered access to primary dental care for children, impacting the quantity of emergency care, reducing restorations of deciduous teeth, and first programmed dental consultations. These findings highlight the need for strategies to ensure that pediatric dental care is not neglected during pandemics.

## Background

Public health systems around the world vary in terms of organization and funding [1]. In Brazil, the Unified Health System (SUS) is a universal healthcare system funded by public funds and managed in a decentralized manner by states and municipalities [2]. The SUS is organized into three levels of healthcare, ensuring that the population receives care according to their specific needs and ensuring continuity of care at all levels [3].

In terms of Primary Health Care (PHC), Brazil offers a range of dental services for children, including the application of fluoride, dental sealants, restorations of deciduous and permanent teeth, prevention and treatment of cavities, clinical exams [4, 5], oral hygiene guidance, and referrals for more specific treatments when necessary [6]. These procedures are crucial in ensuring children’s oral health [7, 8], preventing problems such as early loss of teeth, which can affect speech development [9], chewing [10], and quality of life [11].

However, the COVID-19 pandemic has brought significant changes to dental care [12], including the interruption of elective procedures to avoid gatherings of people within healthcare facilities, the adoption of strict safety measures to prevent the spread of the virus, such as patient screening, telemedicine, the use of personal protective equipment, and rigorous disinfection of workspaces [13, 14].

Despite the importance of these measures, the reduction in the availability of dental services has significantly affected population access [15–19]. In fact, in the country, during the exponential growth of infections in April 2020, pediatric dental procedures suffered a drastic reduction of 89% [20]. It is worth noting that the lack of care can worsen cases of pain, infections, and other pathologies, increasing the demand for emergency services and reducing the effectiveness of oral disease prevention and control programs [21].

It is important to remember that before the COVID-19 pandemic, the oral health conditions of children were already a concern, with considerable dental caries experience [22]. Furthermore, the transition from deciduous to permanent teeth, which occurs between six and twelve years of age, should be monitored by professionals, and accompanied by guidance on healthy eating and oral hygiene [23].

In light of the above, it is necessary to understand and monitor the impact of the pandemic on public dental care to plan and organize the provision of oral health services [24], especially for children. Considering the possibility of various clinical procedures for prevention and treatment being carried out in primary healthcare in Brazil, this study focused on the coverage of the first programmed dental consultation, restoration of permanent and deciduous teeth, individual topical fluoride application per session, emergency care, and deciduous tooth extraction as the most frequent dental procedures for children aged 6 to 12 years. The present study aims to analyze these indicators during the pre-pandemic period (March 1, 2019, to February 29, 2020) and throughout the COVID-19 pandemic (April 1, 2020, to March 31, 2021in a Brazilian region.

## Methods

### Study design

This is an ecological, analytical epidemiological study utilizing secondary data. The methodological design adhered to the theoretical references in the literature [25–27].

### Research scenario

The research was conducted in the municipality of Piracicaba, São Paulo, Brazil, which boasts a population of approximately 410,000 inhabitants [28]. The comprehensive public health network in the municipality includes 51 Family Health Strategy (ESF) units, 20 Basic Health Units (UBS), a center for medical specialties, two centers for dental specialties, four units for emergency medical care, one unit for emergency orthopedic and traumatology care, one polyclinic, and two reference hospitals, as of 2021. The public dental service in primary healthcare is comprised of 30 ESF UBS and 17 UBS modality I (dentist and assistant). In this study, units were chosen based on their adherence to criteria for consistent and ongoing oral health care throughout the assessed periods.

### Data collection

The study collected data from 20 UBS (Basic Health Units) using the OLOSTECH system, a meticulous recording system for various dental health procedures. OLOSTECH, a management software widely employed throughout the municipality of Piracicaba, captures and documents all primary care procedures carried out by healthcare professionals. Functioning as a comprehensive recording, accounting, and storage platform, OLOSTECH ensures the systematic organization of this information. Notably, access to the data inputted into OLOSTECH is restricted, limited to coordinators from each health area, safeguarding its confidentiality. The recorded procedures include the initial scheduled dental consultation, restoration of permanent and deciduous teeth, individual topical fluoride application per session, emergency care, and extraction of deciduous teeth. The collected data were then consolidated into two distinct periods: Period I (March 1, 2019, to February 29, 2020), and Period II (April 1, 2020, to March 31, 2021). These periods represent the timeframes before and during the COVID-19 pandemic in Brazil, respectively. The data was collected from August 2022, after the data was closed by the management tool used in the municipality. Only the procedures carried out in relation to the age group of 6 to 12 years old were collected and evaluated.

### Data analysis

Initially, descriptive and exploratory analyses of the data were performed. Results were described with means, standard deviations, and quartiles. Since the data did not meet the assumptions of parametric analysis, comparisons between the periods before and during the pandemic were made using the paired Wilcoxon nonparametric test. All analyses were performed using the R program with a significance level of 5%. (R Core Team, 2022).

### Ethical aspects

Data access was requested from the manager in charge of the municipality’s health information system. The release of the data was granted subject to the anonymization of participants’ personal data and approval by the ethics committee. No individual data was collected from the children assessed. The study was approved by the Research Ethics Committee of the Piracicaba Dental School - Unicamp (CAAE: 52283621.7.0000.5418).

## Results

Data from 19 complete UBS were collected. In the 12 months prior to the pandemic, the UBS provided 2,015 dental services to children aged 6 to 12 years. In the first 12 months of the pandemic, the same UBS provided 1,659 services to this age group, representing a decrease of 356 services (17.7%) in 12 months.

**Fig. 1:**
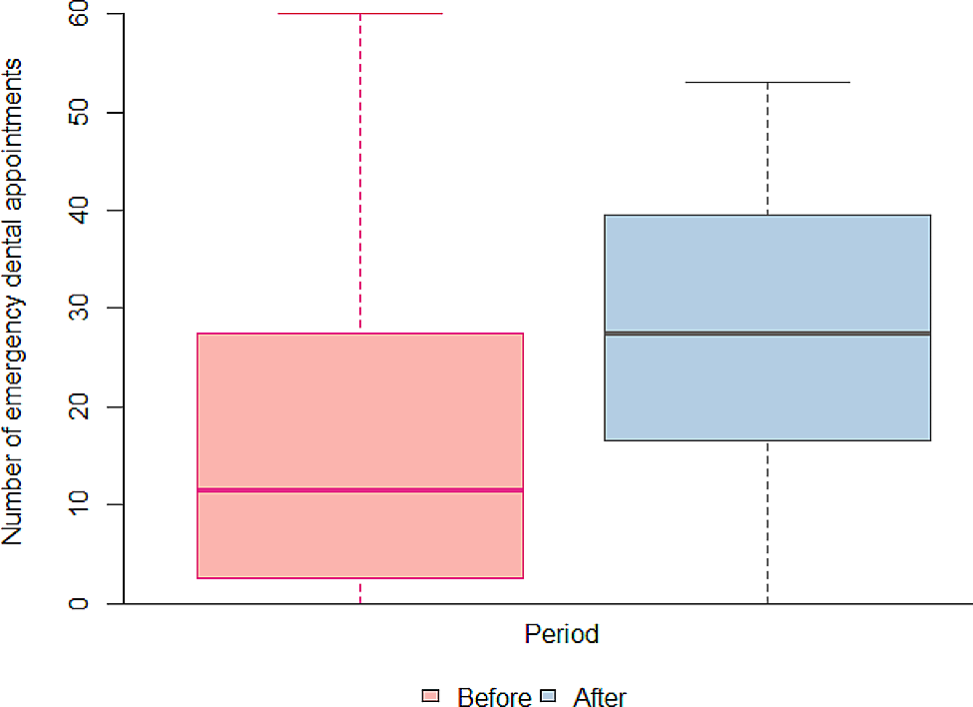
Box plot of the number of emergency dental appointments performed in children aged 6 to 12 years, before and during the COVID-19 pandemic, in the Family Health Units of Piracicaba, SP, *n* = 19. Before: March 1, 2019, to February 29, 2020; During: April 1, 2020, to March 31, 2021

**Fig. 2:**
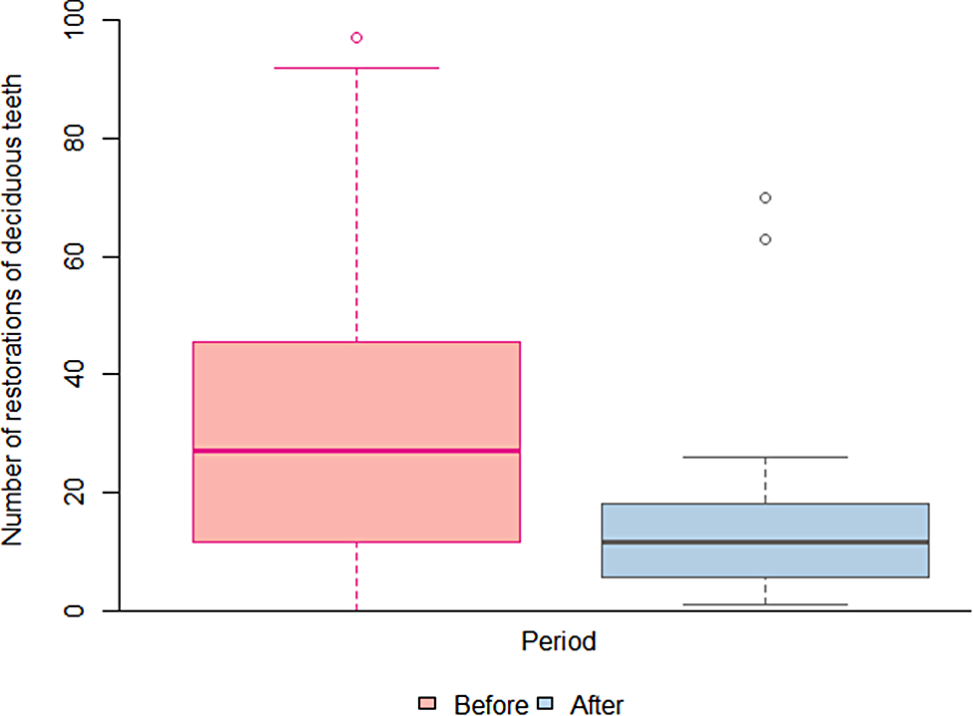
Box plot of the number of restorations of deciduous teeth performed in children aged 6 to 12 years, before and during the COVID-19 pandemic in the Family Health Units of Piracicaba, SP, *n* = 19. Before: March 1, 2019 to February 29, 2020; During: April 1, 2020 to March 31, 2021

Table 1 shows that the number of emergency services increased significantly during the pandemic, *p* < 0.05 (Fig. 1). The number of emergency services provided by the 19 UBS increased from 311 before the pandemic to 537 during the pandemic, computed in 12 months. There was also a significant decrease in the number of restorations of deciduous teeth (*p* < 0.05), as shown in Fig. 2.

**Table 1:** Results of the analysis of the number of dental procedures performed in children aged 6 to 12 years, before and during the COVID-19 pandemic, in the Family Health Units of Piracicaba, SP, Brazil

Procedure	Period	Average (standard deviation)	Minimum	First quartile	Median	Third quartile	Maximum	Total appointments in 19 Units (% of total in period)
Topical fluoride application	Before	8.4 (10.5)	0.0	0.8	2.5	14.2	31.0	167 (8.3%)
	During	7.6 (15.9)	0.0	0.0	2.0	5.0	67.0	153 (9.2%)
*p*-value		0.4899						
Emergency care	Before	15.6 (15.6)	0.0	2.8	11.5	27.2	60.0	311 (15.4%)
	During	26.8 (15.5)	0.0	16.8	27.5	39.2	53.0	537 (32.4%)
*p*-value		0.0095						
Deciduous tooth extraction	Before	9.2 (7.6)	0.0	3.8	7.0	13.0	25.0	183 (9.1%)
	During	11.8 (7.8)	0.0	6.2	10.5	18.2	24.0	236 (14.2%)
*p*-value		0,3869						
First consultation	Before	29.8 (22.9)	2.0	9.8	24.5	47.2	70.0	597 (28.7%)
	During	16.6 (20.7)	0.0	2.8	5.5	25.2	65.0	333 (20.1%)
*p*-value		0,0930						
Deciduous tooth restoration	Before	33.0 (29.5)	0.0	13.8	27.0	45.2	97.0	660 (32.8%)
	During	16.8 (18.4)	1.0	5.8	11.5	17.5	70.0	335 (20.2%)
*p*-value		0,0217						
Permanent tooth restoration	Before	4.8 (4.7)	0.0	1.0	3.5	8.0	17.0	97 (4.8%)
	During	3.2 (3.3)	0.0	0.8	3.0	5.0	13.0	65 (3.9%)
*p*-value		0,1298						
Total	Before	100.8 (61.1)	6.0	49.2	104.5	149.5	210.0	2015 (100.0%)
	During	83.0 (60.3)	5.0	49.2	70.5	92.0	235.0	1659 (100.0%)
*p*-value		0.2043						

Before: March 1, 2019 to February 29, 2020; During: April 1, 2020 to March 31, 2021

**Fig. 3:**
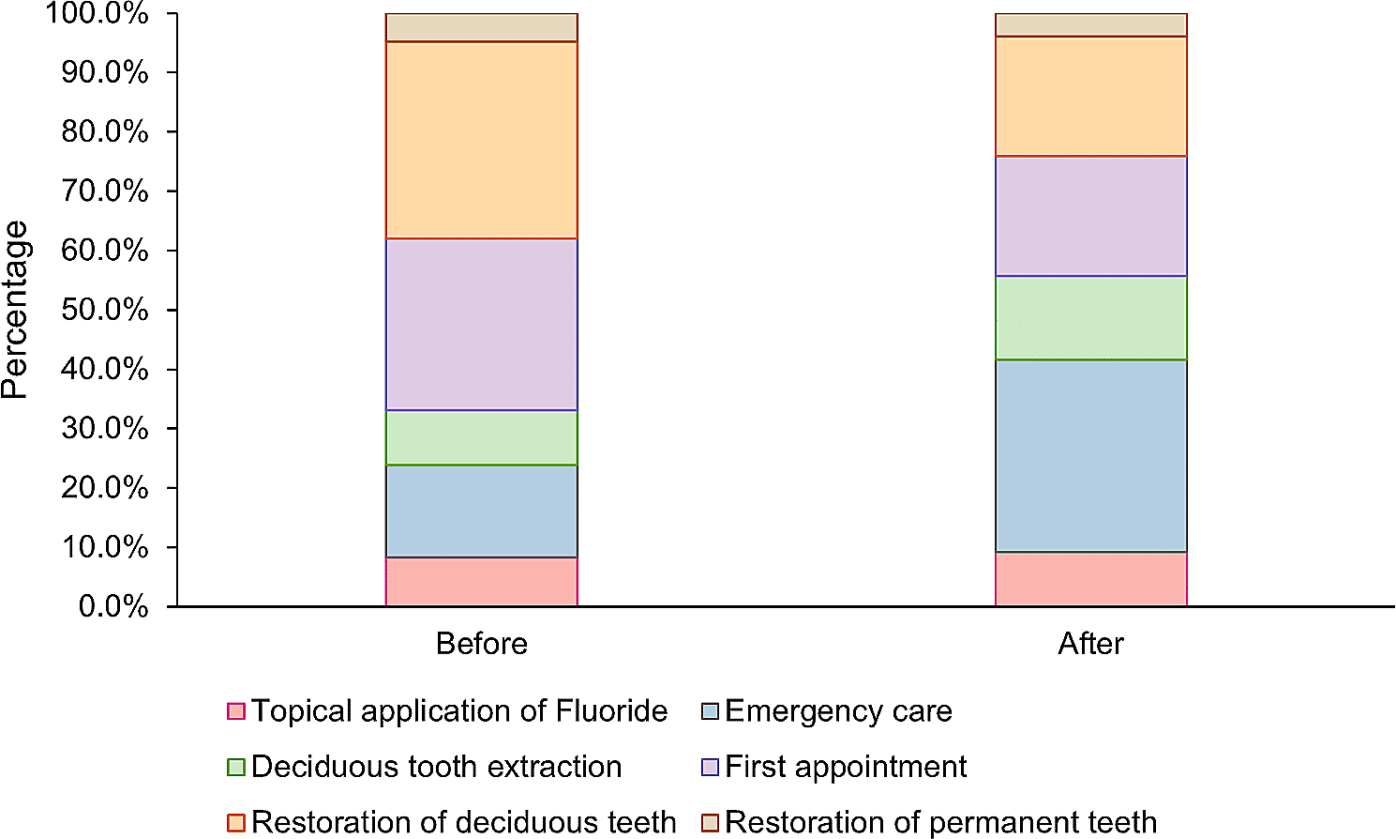
Distribution of dental procedures performed in children aged 06 to 12 years, before and during the COVID-19 pandemic in the Family Health Units of Piracicaba, SP, *n* = 19. Before: March 01, 2019 to February 29, 2020; During: April 01, 2020 to March 31, 2021

The number of restorations decreased from 660 before the pandemic to 335 during the pandemic. Table 1; Fig. 3 present the frequency distribution of procedures before and during the pandemic. The emergency services grew from 15.4% of total services before the pandemic to 32.4% during the pandemic, while the number of restorations of deciduous teeth decreased from 32.8% of services before the pandemic to 20.2% during the pandemic.

## Discussion

This study analyzed the number of dental procedures performed in PHC, aimed at children aged 06 to 12 years old in a Brazilian municipality, comparing two periods, before and during the COVID-19 pandemic. The results showed a significant change in the number of emergency procedures and restoration of deciduous teeth, as well as a significant reduction in the number of first consultation appointments.

Similarly, at the national level, dental procedures (restoration, tooth extraction, and endodontics in deciduous teeth) directed at children in PHC decreased by 66% between January 2019 and May 2020 [20]. Due to the high transmissibility and lethality of the new coronavirus, many changes had to be made in the dental environment [29, 30]. Elective procedures were suspended, and dental professionals were incorporated to support frontline healthcare workers [31]. This scenario probably contributed to the reduction in the number of dental appointments and procedures performed.

An increase in emergency appointments during the pandemic period was particularly noteworthy. A study that considered the entire Brazilian territory identified a 72% decrease in emergency dental treatment in PHC, before and after the COVID-19 pandemic. The research also revealed that the richer regions of the country (South and Southeast) were less affected than the more vulnerable regions, highlighting inequalities in access to primary services [15].

In fact, comparing the months of March to June 2019 and 2020, municipalities with a higher Human Development Index and greater coverage of PHC presented a lower reduction in emergency appointments at this level of care [32]. Therefore, it can be considered that living conditions and service structuring in the study’s scenario may have helped children access emergency treatments.

The literature reports that the most common emergency dental problems before and after the COVID-19 pandemic were acute infections and dental traumas [30]. In the municipality investigated, out of the total of 1,228 emergency appointments, it was identified that the main reason for the dental consultation was medication for acute pain relief (32.6%) [29]. Thus, it is important to note that pain control, provided by emergency appointments, may be due to a pharmacological approach aimed at avoiding the formation of aerosols [15]. In the long run, these cases should be reevaluated. The concern to reassess and monitor emergency cases, coupled with the increased self-reported dental pain during the COVID-19 pandemic [33], points to an increase in demand in the area of oral health and the need to organize primary services to meet these needs.

The same concern arises when the results show a decrease in restoration procedures in deciduous teeth. In a state in southern Brazil, there was a 61.7% reduction in procedures in deciduous teeth when compared to the pre-pandemic period (2018–2019) and the pandemic period (2020–2021). For example, the number of restoration procedures in deciduous teeth decreased from 94 to 34% [34]. It is worth noting that the lack of restorative interventions can lead to dental caries, pain, and premature loss of teeth, impairing occlusion and consequently the quality of life of children [35].

At a time when the prevention of dental diseases should be imperative, especially in the transition phase between deciduous and permanent dentition, there was an inversion of primary dental care, observed by the increase in emergency appointments, and the reduction of first programmatic dental consultations.

The first programmed dental consultation is an individual procedure performed in primary care, aimed at evaluating general oral health conditions, including clinical examination and development of a preventive and/or therapeutic plan [36]. In Brazil, the first programmed dental consultation has decreased by around 42% between January/April 2019 and the same period in 2020 [19], corroborating with the results. This reveals that preventive care was not offered much during the analyzed period. In this sense, the use of tele-dentistry could be reinforced, assisting in the assessment of needs and providing guidance to minimize the reduction of in-person appointments [18]. Although this study evaluated a municipality in Brazil, the results can serve as initial guidance for improvements in primary services. Two important implications were discovered. The first is that the COVID-19 pandemic significantly affected dental procedures for children in primary care, especially restorative treatments and first programmed consultations. The second is that primary services were unable to ensure access to the prevention of oral conditions during periods of health crisis. Considering the consequences of oral problems throughout life, even in extreme scenarios, public health policies should protect children by ensuring necessary treatments.

Additionally, further studies should be conducted to understand the effects of reduced access in the long term. One limitation of this study is its ecological nature, which is unable to establish a cause-and-effect relationship between variables. Additionally, there may be a bias due to the aggregation of data at the population level, without considering individual variability [37]. Another limitation is the source of data acquisition. The use of secondary data from the public service information system, during a tumultuous period of workload overload and human resource deficit, may have contributed to the lack of data records. However, these data are official and used by the municipality for financial resource allocation. Other significant limitation is the absence of data on health education procedures, application of sealants, evidence of dental biofilm, application of sealants and cariostatics per tooth, atraumatic restorative treatments, and the use of minimal intervention. Additionally, the study did not capture information on telemonitoring consultations. These activities are integral to maintaining children’s oral health, and their exclusion represents a limitation in comprehensively understanding the broader landscape of pediatric dental care during the pandemic.

Finally, the COVID-19 epidemic had impacts on primary dental procedures. The essential need for continuous reorganization and adaptation of oral health services is underscored, given the swift changes brought about by COVID-19. Professionals involved in these services should undergo training to ensure both individual and collective protection. It is recommended that managers coordinate efforts to provide support for emerging demands and implement comprehensive measures for the prevention and control of oral health issues. Additionally, facilitating access to elective services for future dental treatments is suggested.

## Conclusion

There was a significant impact on primary dental procedures during the COVID-19 pandemic, specifically those directed towards children in the transition phase from deciduous to permanent teeth. There was a considerable increase in emergency visits and a significant reduction in procedures related to the restoration of deciduous teeth and the initial dental examination.

## Data Availability

The datasets used and/or analysed during the current study are available from the corresponding author on reasonable request.

## Abbreviations

SUS: Unified Health System
UBS: Basic Health Units
ESF: Family Health Strategy
PHC: Primary Health Care
